# Multi-Omics Analysis of PBMCs Revealed Distinct Biological Differences Between Hemodialysis and Peritoneal Dialysis

**DOI:** 10.3390/ijms27167470

**Published:** 2026-08-20

**Authors:** Maurizio Bruschi, Simona Granata, Silvia Lai, Valentina Pistolesi, Laura Barberio, Rossana D’Agostino, Francesca Sorace, Francesca Giordano, Paola Pontrelli, Loreto Gesualdo, Lida Tartaglione, Giovanni Candiano, Sonia Spinelli, Andrea Petretto, Chiara Lavarello, Edoardo La Porta, Michele Provenzano, Gianluigi Zaza

**Affiliations:** 1 Laboratory of Molecular Nephrology, Istituto di Ricovero e Cura a Carattere Scientifico (IRCCS) Istituto Giannina Gaslini, 16147 Genoa, Italy; maurizio.bruschi@unige.it (M.B.); giovanni.candiano.54@gmail.com (G.C.); soniaspinelli@gaslini.org (S.S.); 2 Department of Experimental Medicine (DIMES), University of Genoa, 16126 Genoa, Italy; 3 Department of Biology, Ecology and Earth Sciences, University of Calabria, 87036 Rende, Italy; simona.granata@unifg.it (S.G.); laura.barberio@unical.it (L.B.); rossana.dagostino@unical.it (R.D.); francesca.sorace@unical.it (F.S.); 4 Nephrology, Dialysis and Transplantation Unit, Policlinico Riuniti University Hospital, 71122 Foggia, Italy; 5 Nephrology, Dialysis and Transplantation Unit, Department of Translational and Precision Medicine, Sapienza University of Rome-Policlinico Umberto I, 00185 Rome, Italy; silvia.lai@uniroma1.it; 6 Nephrology, Dialysis and Transplantation Unit, Policlinico Umberto I, 00161 Rome, Italy; valentina.pistolesi@uniroma1.it (V.P.); lida.tartaglione@uniroma1.it (L.T.); 7 Department of Pharmacy, Health and Nutritional Sciences, University of Calabria, 87036 Rende, Italy; francesca.giordano@unical.it (F.G.); michele.provenzano@unical.it (M.P.); 8 Nephrology, Dialysis and Transplantation Unit, Department of Precision and Regenerative Medicine and Ionian Area (DiMePRe-J), University of Bari Aldo Moro, 70124 Bari, Italy; paola.pontrelli@uniba.it (P.P.); loreto.gesualdo@uniba.it (L.G.); 9 Core Facility for Omics Sciences, IRCCS Istituto Giannina, 16147 Genoa, Italy; andreapetretto@gaslini.org (A.P.); chiaralavarello@gaslini.org (C.L.); 10 Nephrology, Dialysis and Transplantation Unit, Istituto di Ricovero e Cura a Carattere Scientifico (IRCCS) Istituto Giannina Gaslini, 16147 Genoa, Italy; edoardolaporta@gaslini.org; 11 Division of Nephrology, Dialysis and Transplantation, Annunziata Hospital, 87100 Cosenza, Italy

**Keywords:** hemodialysis, peritoneal dialysis, proteomics, chronic kidney disease, cellular senescence, IRF8

## Abstract

Although the clinical differences between hemodialysis (HD) and peritoneal dialysis (PD) are well established, the specific biological mechanisms underlying these differences remain incompletely understood. We performed untargeted proteomic and transcriptomic analyses of peripheral blood mononuclear cells (PBMCs) from healthy controls (CTRs) and patients with chronic kidney disease undergoing HD or PD. Key findings were validated in an independent cohort using standardized biomolecular techniques. Bioinformatic analysis identified 36 differentially expressed proteins and 19 transcripts between HD and PD according to the predefined FDR-adjusted significance threshold. Functional enrichment analysis revealed that these factors were primarily involved in cellular senescence, aging, and stress-response pathways. Among the identified molecules, interferon regulatory factor 8 (IRF8) was the only factor consistently upregulated in HD compared with PD in both omics analyses. This finding was confirmed by ELISA in an independent cohort (*p* < 0.0001). Kinase enrichment analysis prioritized Checkpoint Kinase 2 (CHEK2), which was also identified by proteomics, as a predicted upstream regulator and warrants further investigation. Overall, these findings identify distinct molecular signatures in PBMCs from HD and PD patients and suggest a potential association between HD and increased PBMC IRF8 abundance and a senescence-related signature, which requires validation in larger and longitudinal cohorts.

## 1. Introduction

Chronic kidney disease (CKD) is a global health burden that affects an estimated 10–13% of the world population [1,2]. The disease is characterized by a progressive and irreversible decline in renal function driven by multiple pathological mechanisms, including chronic inflammation, oxidative stress, endothelial dysfunction, and metabolic dysregulation [3,4,5]. The clinical course of CKD is frequently complicated by a markedly increased risk of comorbidities, most notably cardiovascular disease and infections, which impact the prognosis of patients [6,7]. In addition, patients with CKD exhibit a significantly higher rate of all-cause mortality than the general population. A central contributor to these adverse outcomes is the progressive accumulation of uremic toxins and metabolic waste products resulting from impaired renal clearance. These retained solutes exert systemic toxic effects, promoting immune dysfunction, vascular damage, and organ injury, thereby accelerating disease progression and worsening CKD-associated complications [8,9].

In the final stage of CKD (stage 5, end-stage renal disease), severe metabolic imbalance necessitates the initiation of renal replacement therapy, including hemodialysis (HD) or peritoneal dialysis (PD). However, despite substantial advances in the biocompatibility of HD membrane materials and PD fluid over recent decades, both dialysis modalities remain frequently associated with significant complications driven by profound systemic biological alterations.

In this context, activated peripheral blood mononuclear cells (PBMCs) play a central pathogenic role by triggering multiple biological processes, such as inflammation, oxidative stress, epithelial- and endothelial-to-mesenchymal transition-associated fibrosis, and neoangiogenesis, both within multiple organs and tissues (systemic effects) and in the mesothelial cavity (local effects) [10].

During HD, the interaction of blood with the artificial dialysis membrane activates circulating immune cells, resulting in the release of a broad spectrum of proinflammatory cytokines and mediators. This immune activation promotes a persistent state of chronic low-grade inflammation and oxidative stress, which in turn contributes to protein–energy wasting, endothelial dysfunction, and vascular remodeling [11,12].

In contrast, during PD, chronic exposure to conventional PD solutions, characterized by the presence of plasticizers, high glucose concentrations, glucose degradation products, low pH, and high osmolality, can induce significant peritoneal functional alterations. These changes include loss or degeneration of mesothelial cells, submesothelial thickening, and vascular abnormalities. Moreover, mesothelial injury promotes the recruitment and activation of inflammatory cells, leading to the production of pro-inflammatory cytokines and hepatocyte growth factors, as well as an imbalance between pro-oxidant and antioxidant mechanisms, resulting in elevated oxidative stress and immune system dysregulation [13,14]. Collectively, these processes contribute to both acute and chronic local complications, such as peritoneal membrane failure and peritonitis, and accelerate the development of systemic comorbidities.

Although the different clinical impacts of HD and PD are well documented, the biological mechanisms underlying these disparities remain incompletely understood and represent an active area of investigation. While omics technologies have significantly expanded our understanding of CKD-associated molecular alterations, the integration of multi-omics approaches provides a more comprehensive and robust framework to unravel the complex biological processes distinguishing between HD and PD. In this study, we performed an integrated proteomic and transcriptomic characterization of PBMCs from patients with CKD undergoing HD or PD. Specifically, we applied an untargeted proteomic analysis to systematically identify molecular signatures associated with dialysis modality and integrated these findings with data from a previously performed transcriptomic analysis, providing novel insights into the pathways potentially underlying the biological differences between HD and PD. The findings obtained from the omics analyses were subsequently validated in an independent cohort of patients using well-established and standardized biomolecular techniques.

## 2. Results

### 2.1. Proteomics and Transcriptomics of PBMCs Discriminate HD from PD Patients

**Figure 1:**
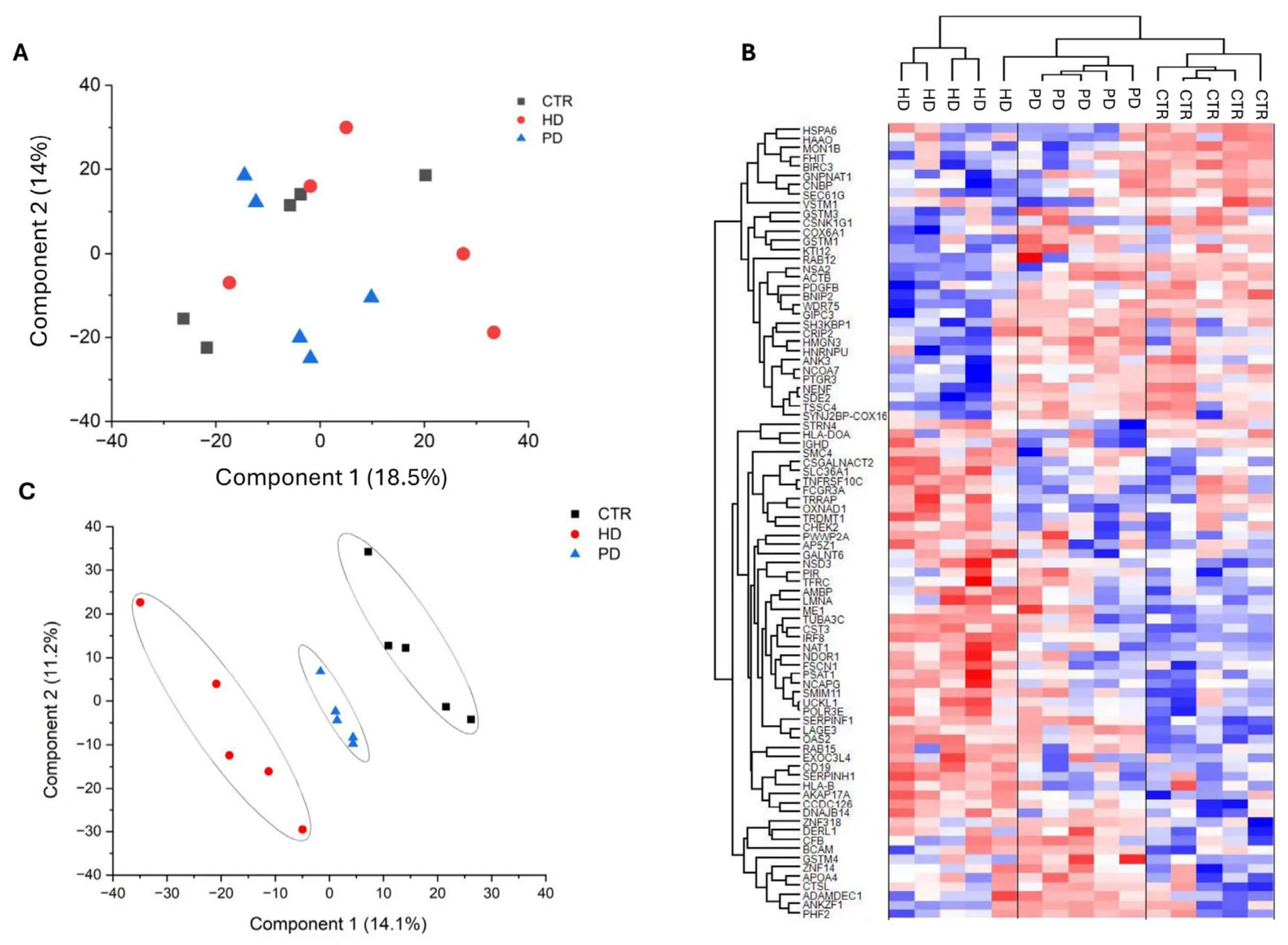
Global and selected-feature analysis of the proteomic dataset. (**A**) Principal component analysis performed using all quantified proteins after data preprocessing, without prior feature selection. (**B**) Heatmap of the 86 proteins showing significant differences in abundance across the three study groups identified by one-way ANOVA. Each row represents a protein, and each column represents a clinical sample. Z-score normalized protein abundances are shown on a pseudocolor scale (red: upregulated, white: mean expression, blue: downregulated). The dendrogram shows the results of the unsupervised hierarchical clustering, grouping samples and proteins based on similarity in abundance profiles. (**C**) PLS-DA plot based on the same statistically significant proteins. Each symbol represents an individual sample, and the ellipses indicate the 95% confidence intervals for each group. K-means clustering identified three distinct clusters corresponding to CTR (black squares), PD (blue triangles), and HD (red circles).

Proteomic analysis identified a total of 5151 proteins. Principal component analysis (PCA), performed using all quantified proteins after data preprocessing, showed no evident outliers and no clear separation according to clinical group (Figure 1A). One-way ANOVA identified 86 proteins that differed significantly among the three groups after the prespecified statistical and bioinformatic filtering steps (Table S1).

Their abundance profiles, visualized after Z-score normalization, are shown in Figure 1B. Partial least squares-discriminant analysis (PLS-DA), performed on the same proteins identified by ANOVA, provided an exploratory supervised visualization of group separation and ranked the proteins according to their Variable Importance in Projection (VIP) scores (Figure 1C). Interestingly, the PD patient cluster was positioned between the HD and CTR groups.

**Figure 2:**
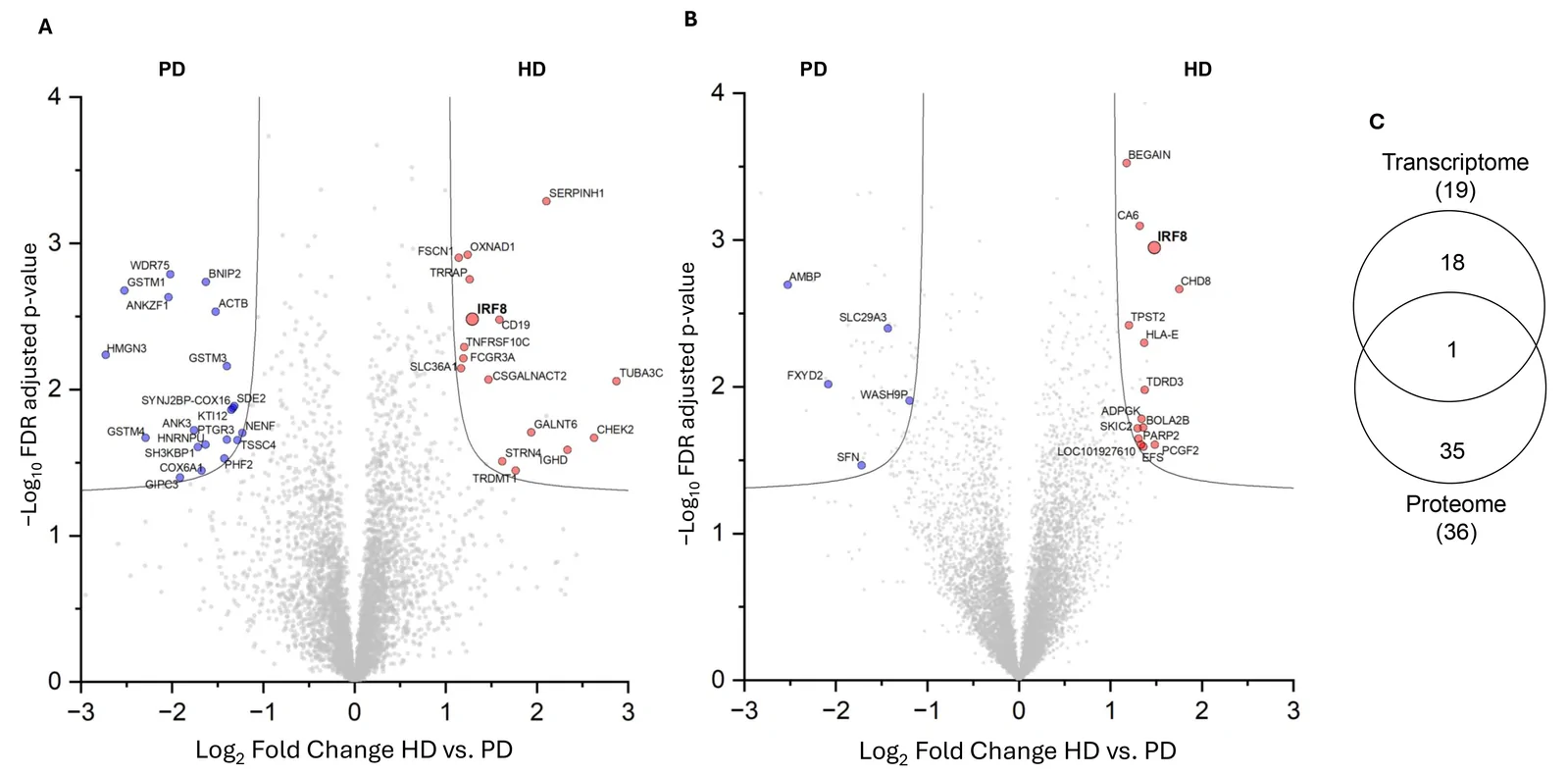
Differentially expressed proteins and transcripts in the HD vs. PD comparison. Volcano plots showing differentially abundant (**A**) proteins and (**B**) transcripts in PBMCs from HD and PD. Red and blue dots indicate proteins or transcripts with statistically significant increased or decreased abundance/expression in HD vs. PD, whereas non-significant features are shown in gray. The black hyperbolic curves represent the significance threshold, corresponding to an absolute log2 fold change ≥ 1 and an FDR-adjusted *p*-value ≤ 0.05. (**C**) Venn diagram showing the overlap and unique statistically significant features identified in the HD versus PD comparison by the proteomic and transcriptomic analyses.

Finally, unpaired *t*-tests were conducted to identify differentially expressed proteins in the pairwise comparisons HD vs. PD (Figure 2A), CTR vs. HD (Figure S1A), and CTR vs. PD (Figure S1B) which identified 36, 41, and 23 significantly discriminative proteins, respectively (Table S2–S4).

Re-analyzing our previously published transcriptomic datasets [15,16] using the same statistical filters applied in the proteomic analysis, we identified 19 genes significantly differentially expressed between HD and PD (Figure 2B).

Among the identified molecules, IRF8 was the only factor consistently upregulated in HD compared with PD at both the proteomic (log_2_ fold change = 1.29, FDR-adjusted *p*-value = 0.0033) and transcriptomic (log_2_ fold change = 1.48, FDR-adjusted *p*-value = 0.0011) levels.

### 2.2. Functional Enrichment Analysis

**Figure 3:**
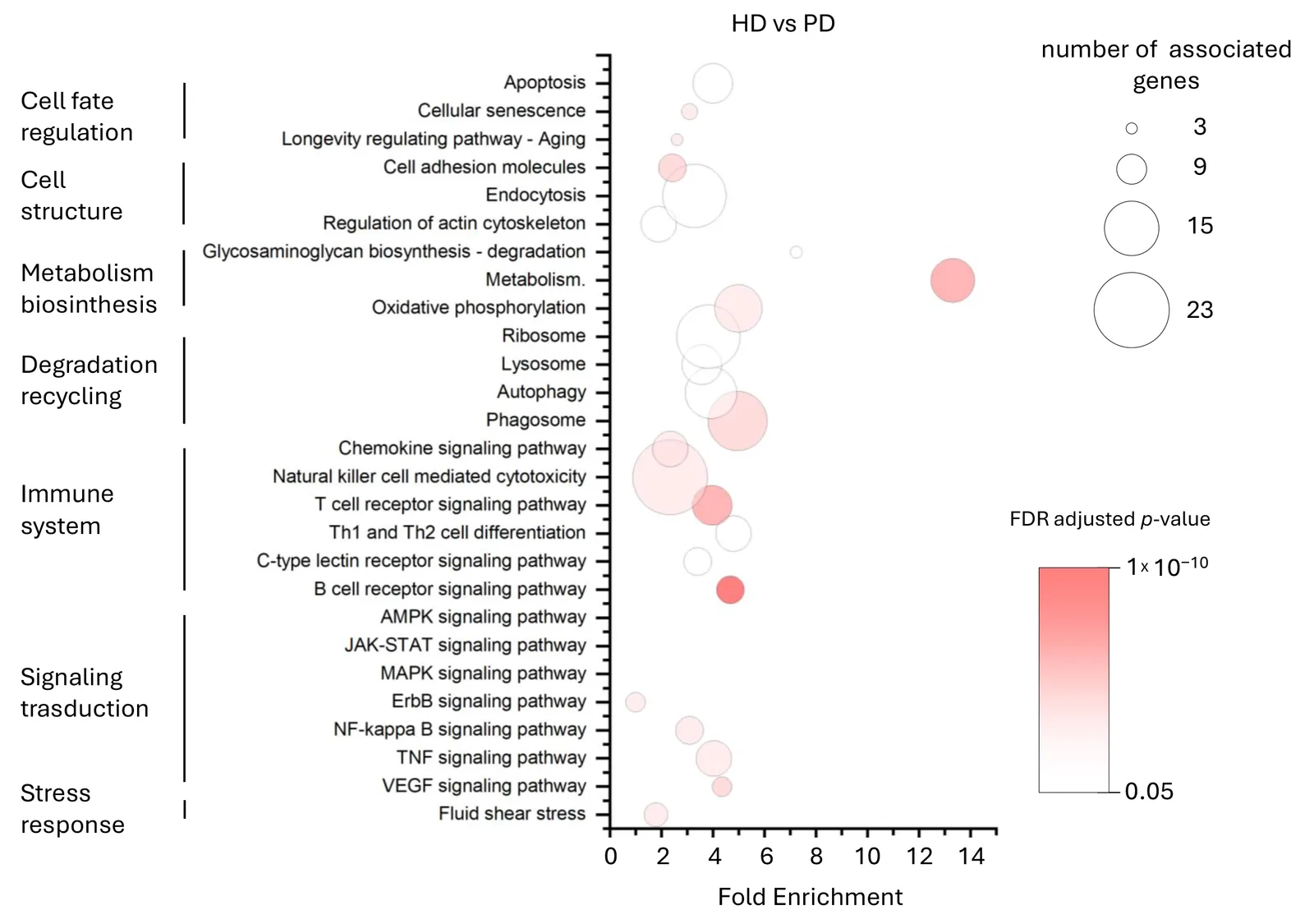
Functional enrichment analysis of the combined set of statistically significant transcripts and proteins differentiating HD and PD. The bubble plot shows the enriched biological processes and pathways identified using the PathfindR package and grouped into seven major functional categories. Enriched terms are displayed on the y-axis. Each circle represents an enriched term; circle size is proportional to the number of associated genes, whereas color indicates the enrichment significance, expressed as FDR-adjusted *p*-value and displayed on a pseudo-color scale ranging from 0.05 (white) to 1 × 10^−10^ (red).

Bioinformatic analysis of the combined set of statistically significant proteins and transcripts from the HD vs. PD comparison identified 27 significantly enriched biological processes and pathways, which were grouped into seven key functional categories: cell fate regulation, cell structure, metabolism and biosynthesis, degradation and recycling, immune system, signal transduction, and stress response (Figure 3). Functional enrichment analysis was performed for all pairwise comparisons (HD vs. PD, HD vs. CTR, and PD vs. CTR), and the complete results are reported in Table S5.

**Figure 4:**
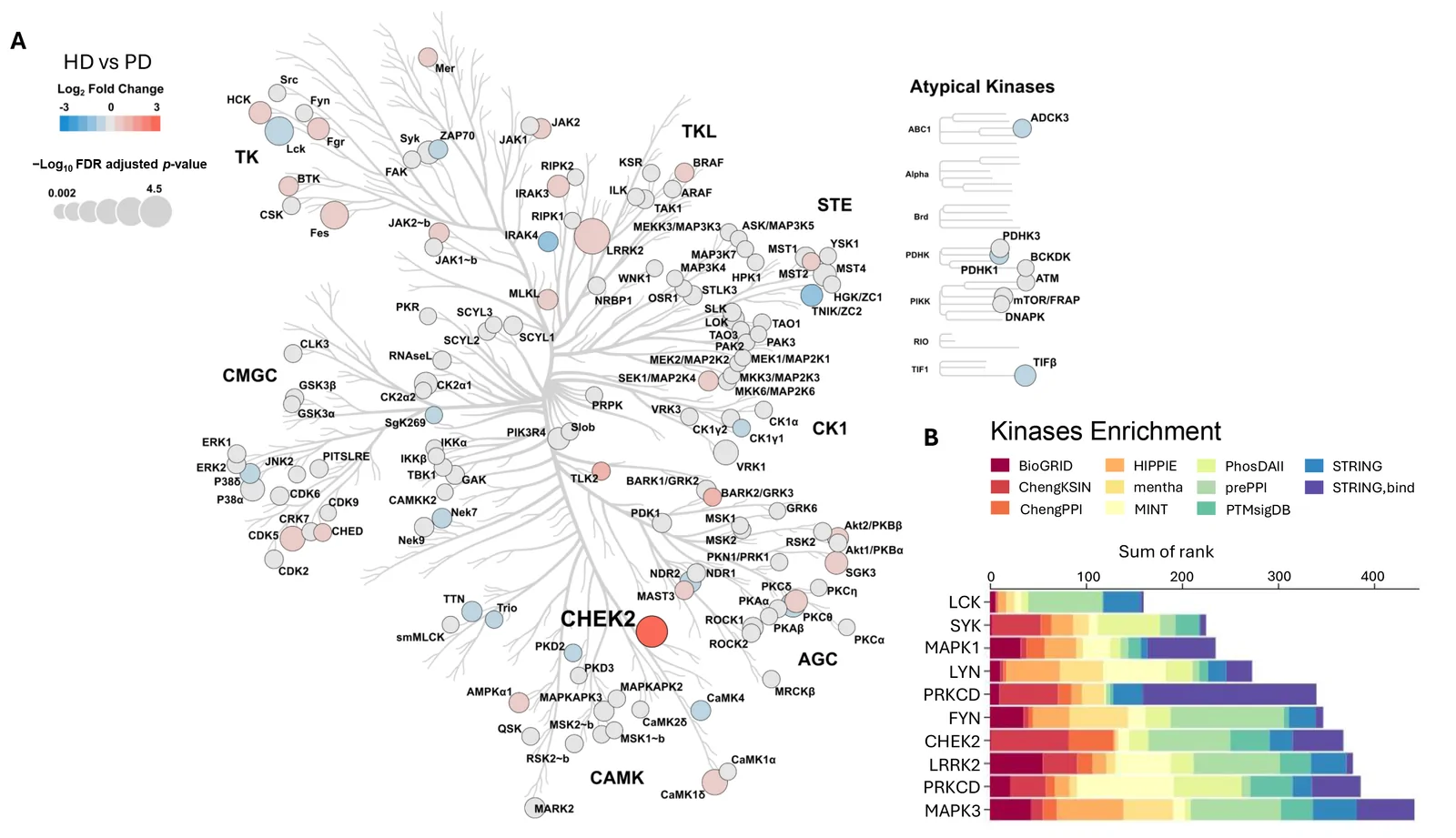
Kinase enrichment analysis. (**A**) Kinome tree showing the differentially expressed kinases identified by proteomic analysis in the HD vs. PD comparison. Each circle represents a kinase, with color and size reflecting the log2 fold change and −log_10_ FDR-adjusted *p*-value, respectively. (**B**) Kinase enrichment analysis performed on the combined set of statistically significant transcripts and proteins identified in the comparison between HD and PD. The plot displays the top 10 predicted upstream kinases ranked by enrichment analysis.

Kinase enrichment analysis was performed on the combined set of statistically significant transcripts and proteins identified in the HD versus PD comparison. Among the top-ranked predicted upstream kinases, CHEK2 was also identified as a differentially expressed kinase in the proteomic dataset (log_2_ fold change = 2.63, FDR-adjusted *p*-value = 0.021) (Figure 4). In addition, CHEK2 was among the proteins associated with enriched biological processes and pathways identified by the functional enrichment analysis (Table S5).

**Figure 5:**
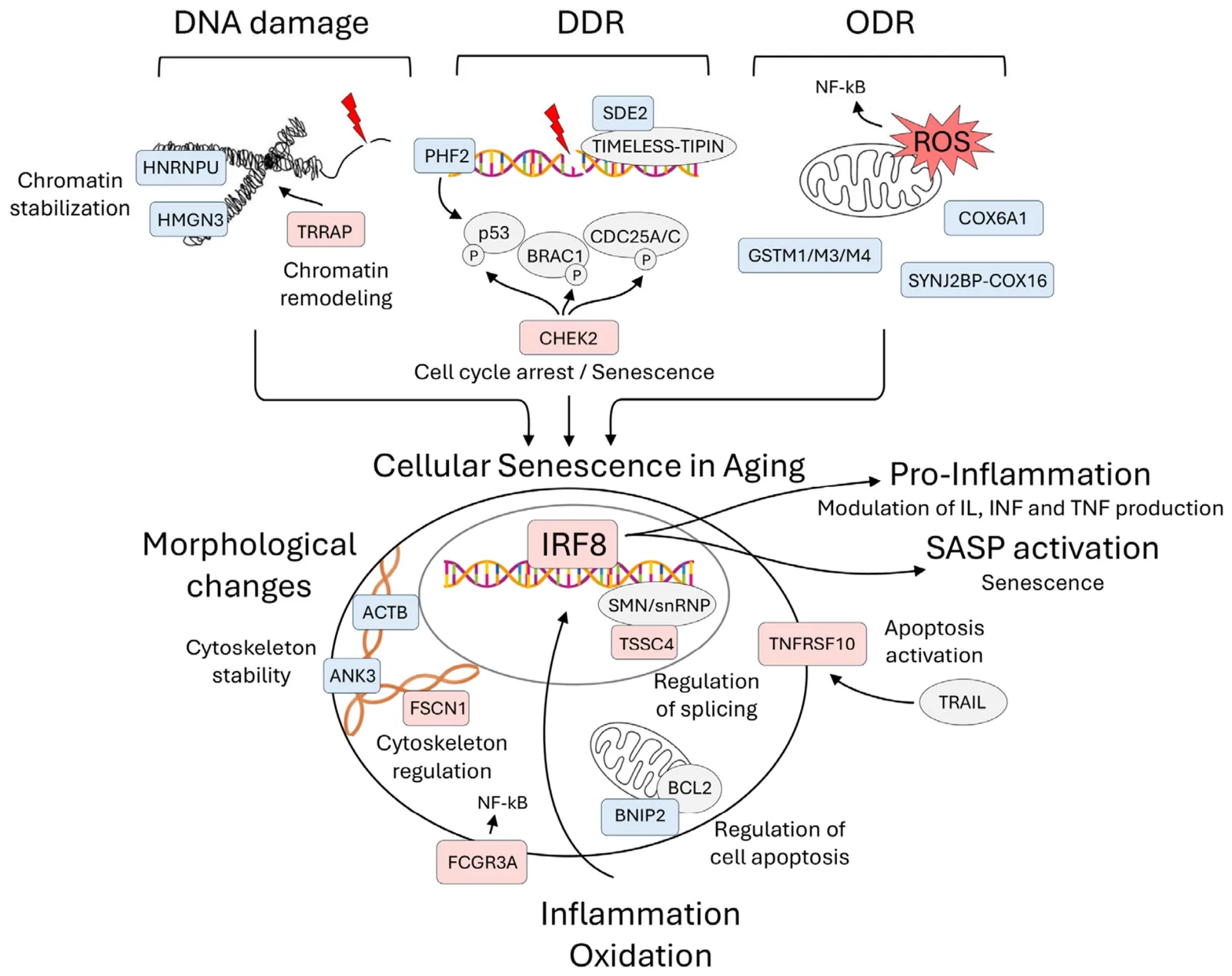
Proposed model of the molecular mechanisms potentially associated with cellular senescence in HD. This diagram depicts a hypothesis-generating model that integrates the results of the proteomic and transcriptomic analyses, functional enrichment analysis, kinase enrichment analysis, and literature. Proteins shown in red and blue were significantly upregulated and downregulated in HD compared to PD, respectively. Protein labels are reported as gene symbols (see Table S1 for protein annotation). Molecules shown in gray and the interactions connecting them represent components inferred from pathway analysis and published literature. Abbreviations: DDR, DNA damage response; ROS, reactive oxygen species; ODR, oxidative damage response; SASP, senescence-associated secretory phenotype.

These findings were integrated into the proposed mechanistic model shown in Figure 5, which summarizes the molecular pathways potentially associated with cellular senescence in HD compared with PD based on the differential expression analyses, functional enrichment analysis, kinase enrichment analysis, and published literature.

### 2.3. Validation of Omics Results by ELISA of IRF8 and Analysis of Senescence in PBMCs from HD Versus PD Patients

**Figure 6:**
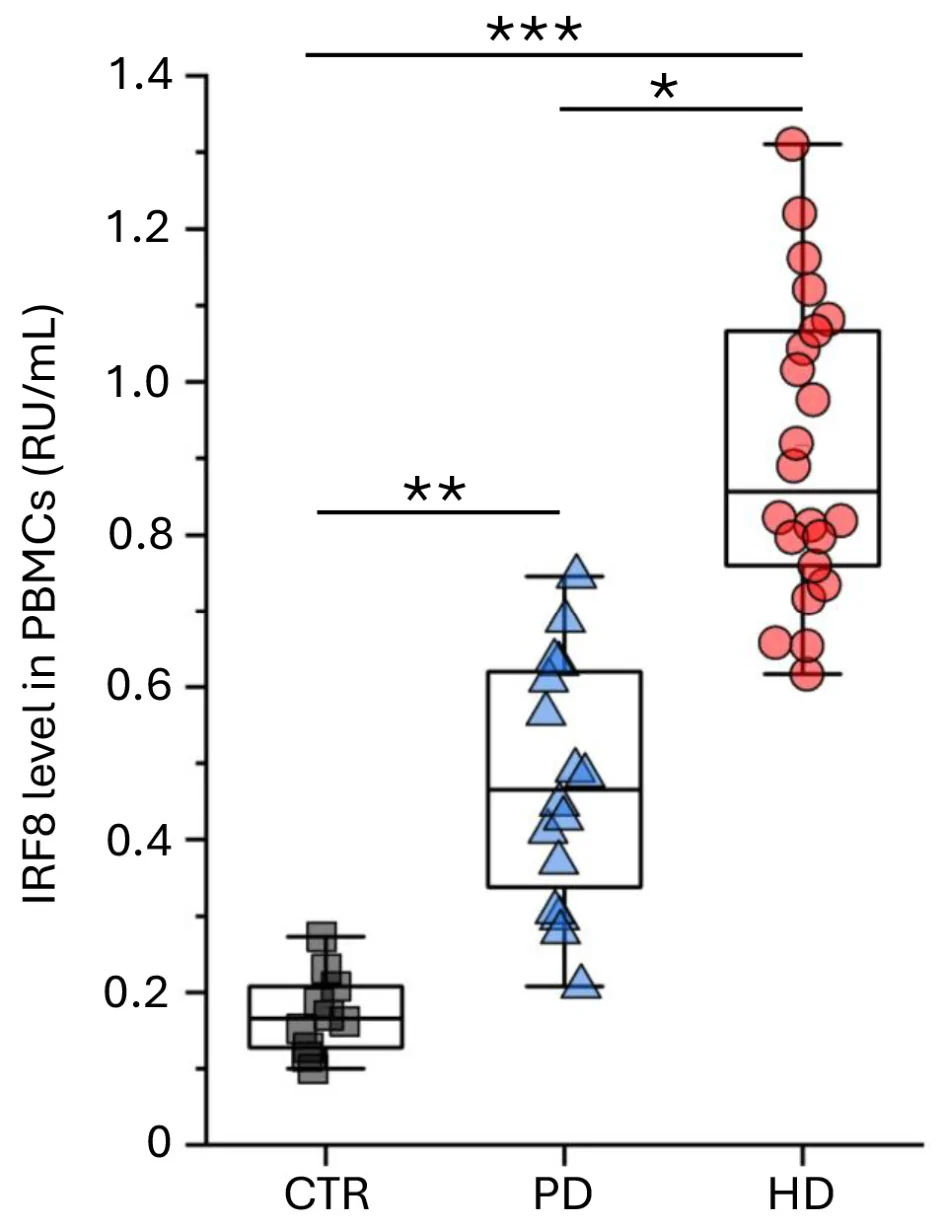
Validation of IRF8 by in-house ELISA. Box plots show IRF8 content in PBMCs from CTR, PD, and HD, measured by the in-house ELISA and expressed as relative units per milliliter (RU/mL). Each data point represents a patient: CTR (grey squares), PD (blue triangles), and HD (red circles). Statistical significance was assessed by Kruskal–Wallis test followed by Benjamini–Hochberg-adjusted post hoc pairwise comparisons. * adjusted *p* < 0.05, ** adjusted *p* < 0.001, and *** adjusted *p* < 0.0001.

To validate the omics findings, we quantified IRF8 levels in PBMCs of the validation cohort using a custom in-house direct ELISA. As shown in Figure 6, IRF8 levels differed significantly among the three groups (Kruskal–Wallis *p* < 0.0001). Post hoc pairwise comparisons with Benjamini–Hochberg adjustment demonstrated significantly higher IRF8 levels in HD patients than in PD patients (adjusted *p* = 1.28 × 10^−4^) and CTR subjects (adjusted *p* = 1.36 × 10^−8^), while PD patients also showed higher IRF8 levels than CTR subjects (adjusted *p* = 1.92 × 10^−2^). The median IRF8 values were 0.86 RU/mL (IQR: 0.75–1.10) for HD patients, 0.46 RU/mL (IQR: 0.32–0.62) for PD patients, and 0.17 RU/mL (IQR: 0.125–0.21) for CTR subjects.

**Figure 7:**
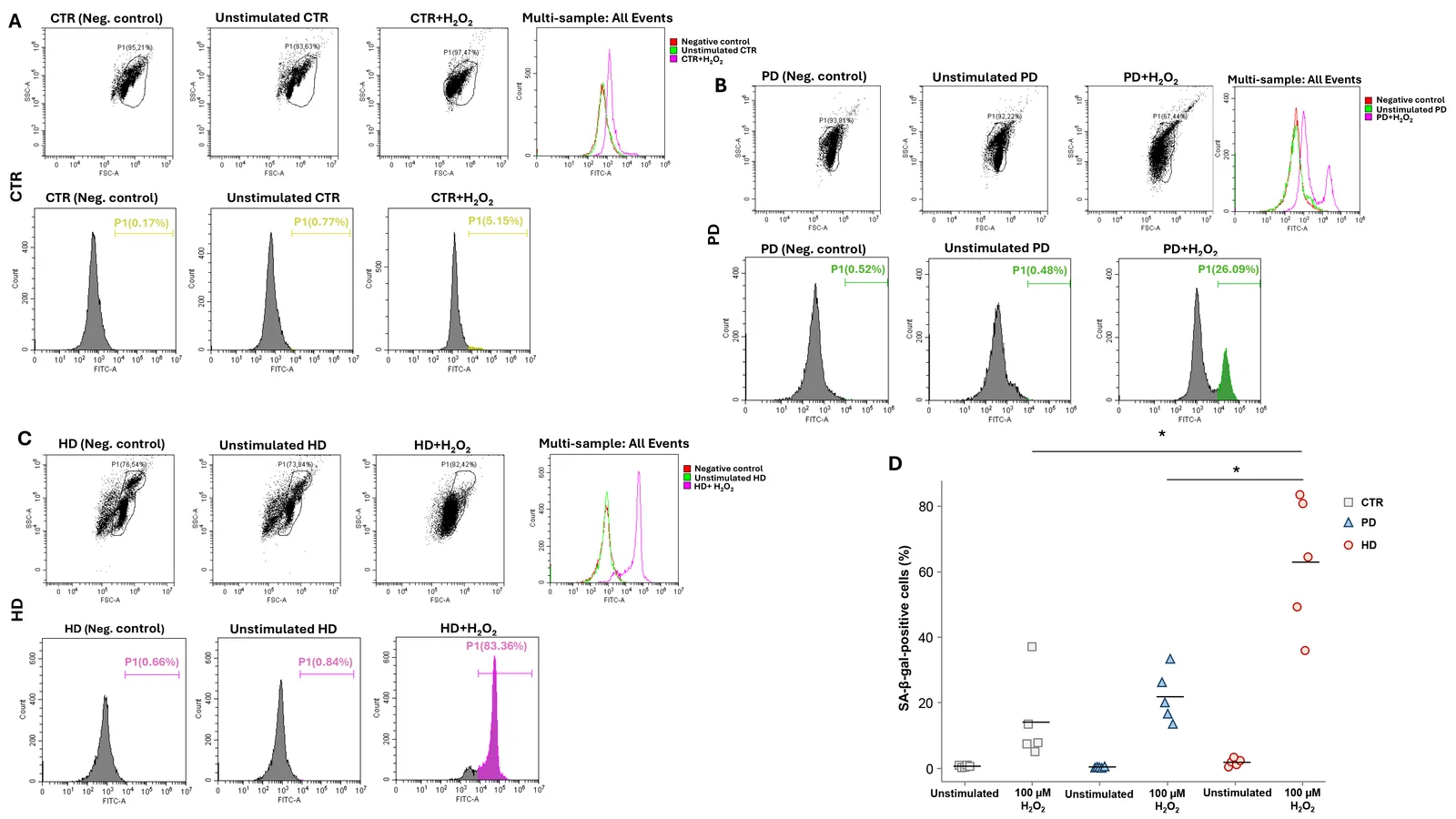
Senescent cells. Representative flow cytometry gating strategy and analysis of β-Gal-positive cells for PBMCs from (**A**) healthy controls (CTRs), (**B**) peritoneal dialysis (PD) patients, and (**C**) hemodialysis (HD) patients, unstimulated or stimulated 18 h with 100 μM H_2_O_2_. Negative control represents the cells incubated with no Cell Event™ Senescence Green probe. (**D**) Dot plot showing the percentage of SA-β-Gal-positive cells in PBMCs from CTR, PD, and HD unstimulated and following 18 h of treatment with 100 μM H_2_O_2_. Data are presented as individual values (*n* = 5 independent experiments). * *p* < 0.05. The bar indicates mean value.

Subsequently, we assessed the number of senescent cells in PBMCs from HD, PD, and CTR after stimulation with 100 μM H_2_O_2_ for 18 h. PBMCs from HD patients exhibited a significantly higher proportion of senescent cells, as indicated by Senescence-Associated β-Galactosidase (SA-β-gal)-positive staining, compared with PD patients and CTRs (*p* < 0.05) (Figure 7). These findings indicate an increased susceptibility of PBMCs from HD patients to stress-induced senescence.

## 3. Discussion

Our study identified a difference in the proteomic profile of peripheral blood mononuclear cells (PBMCs) in patients undergoing maintenance hemodialysis (HD) compared with those undergoing peritoneal dialysis (PD), which could reflect the different impact of these renal replacement therapies on circulating immune cells. In fact, in HD, chronic blood contact with the dialysis devices may directly activate PBMCs, while in PD, their activation may be indirect and related to the continuous interaction of dialysis fluids with the mesothelium [17,18,19].

Bioinformatics identified many differentially expressed proteins involved in several cellular pathways (cell fate regulation, cell structure, metabolism and biosynthesis, degradation and recycling, immune system, signal transduction, and stress response) that differentiated the two study groups. Several enriched biological processes were consistent with mechanisms involved in cellular senescence.

Transcriptomic analysis performed in an independent patient cohort, using robust statistical adjustments, identified 19 genes significantly differentially expressed between HD and PD. Integration with proteomic data revealed that IRF8 was the only biological factor consistently upregulated in both proteomic and transcriptomic analyses in PBMCs from HD compared with PD patients. This multi-omics integration provided complementary insights into the molecular alterations associated with HD and PD. While proteomics reflects changes at the protein level, transcriptomics provides information on gene expression patterns and potential upstream regulatory mechanisms. IRF8 is a transcription factor that plays a critical role in immune cell development and function, particularly in the regulation of monocyte, dendritic cell and Th17, Th9, and Treg differentiation [20,21,22,23]. Beyond its role in immunity, IRF8 has been implicated in processes related to immune aging and cellular senescence [24], particularly damage-induced senescence [25], an important pathway that could be related to major complications in HD patients.

These findings were consistent with the kinase enrichment analysis, which identified CHEK2 among the top-ranked predicted upstream kinases. Notably, CHEK2 was also differentially expressed in the proteomic dataset and was associated with the enriched biological processes and pathways identified by the functional enrichment analysis. CHEK2 is a key component of DNA damage response and has been implicated in the regulation of cellular senescence through DNA damage-induced cell cycle arrest [26]. Therefore, the convergence of the differential expression, kinase enrichment, and functional enrichment analyses suggests that DNA damage response-associated mechanisms may contribute to the molecular differences observed between HD and PD patients.

Taken together, these findings are consistent with accumulating evidence that HD is associated with accelerated biological aging and increased cellular senescence. Previous studies have described several hallmarks of premature aging in HD patients, including chronic low-grade inflammation (inflammaging), T-cell lymphopenia characterized by reduced thymic naïve T-cell output and expansion of CD8^+^ central memory T cells lacking the co-stimulatory molecule CD28, reduced B-cell counts [27], telomere shortening, and activation of DNA damage response pathways [28,29,30,31,32,33,34,35,36].

In particular, enhanced myeloid cell activation, immunosenescence, and sustained pro-inflammatory signaling have been associated with repeated exposure to extracorporeal circulation and oxidative stress during HD [37]. This makes immune cells more vulnerable to additional stress, as evidenced by H_2_O_2_ treatment in PBMCs from HD patients.

Although our findings provide novel insights into the molecular differences between HD and PD, several limitations should be acknowledged. First, the proteomic and transcriptomic analyses were performed using the total PBMC population; therefore, the observed differences, including the increased expression of IRF8, may partly reflect variations in the relative abundance of specific immune-cell subsets between the study groups. Second, while the proteomic findings were supported by transcriptomic data and IRF8 was validated by ELISA, the study was conducted in a relatively small discovery cohort and should be considered hypothesis-generating. Larger independent cohorts are needed to confirm these findings and evaluate the clinical utility of IRF8 as a biomarker. Longitudinal studies are warranted to determine whether IRF8 levels are associated with long-term clinical outcomes and to identify the immune-cell subsets primarily responsible for its increased expression in HD patients.

If confirmed, our observations could support the potential clinical relevance of targeting senescence-associated pathways in kidney disease. In recent years, senolytics and senomorphics agents have proven promising effects in experimental models and are currently undergoing clinical trials [38]. Administration of dasatinib plus quercetin in subjects with diabetic kidney disease attenuated adipose tissue and skin senescent cell burden, decreased adipose tissue macrophage accumulation, and reduced key circulating senescence-associated secretory phenotype (SASP) factors [39]. Similarly, the mTOR-inhibitor rapamycin, administered to a rat model of kidney transplantation in the immediate post-transplant period, reduced the accumulation of p16INK4a-positive cells in tubules, interstitium, and glomerula, decreased SASP factors levels, and reduced monocyte/macrophage and CD8+ T-lymphocyte infiltration in the transplanted kidney [40].

In conclusion, this study demonstrates distinct proteomic and transcriptomic signatures in PBMCs from patients undergoing HD and PD, supporting the concept that different dialysis modalities are associated with distinct molecular responses in circulating immune cells. It also provides additional insights into the molecular mechanisms potentially associated with inflammation and cellular senescence in patients receiving maintenance dialysis. Among the identified molecules, IRF8 may represent a blood-based marker for monitoring systemic pro-aging and senescence-associated processes in patients undergoing maintenance dialysis. CHEK2, identified as both a differentially expressed protein and a top-ranked predicted upstream kinase, warrants further investigation as a potential component of these molecular pathways. However, at present, the lack of large-scale analyses evaluating diagnostic performance and associations with clinical outcomes does not allow us to consider it a biomarker ready for clinical application.

Senescence-associated immunological changes in HD predispose patients to cardiovascular and infectious complications, adversely affecting clinical outcomes and mortality [41]. Therefore, a deeper understanding of the molecular mechanisms underlying these processes may facilitate the development of strategies to attenuate immunological senescence and improve patient outcomes.

## 4. Materials and Methods

### 4.1. Patients

A total of 63 subjects were enrolled in the study after providing informed consent in accordance with the Declaration of Helsinki. Subjects were assigned to a discovery group (n = 15) or a validation group (n = 48).

Discovery Proteomic Group: This group was used for proteomic analysis and included 5 healthy controls (CTRs), 5 peritoneal dialysis (PD) patients, and 5 hemodialysis (HD) patients.

Validation Group: This group was used to confirm the findings of the discovery group and included 10 CTRs, 16 PD patients, and 22 HD patients.

Data from the transcriptomic analysis we previously performed in PBMCs from 5 CTRs, 10 PD patients, and 17 HD patients [15,16] were also used (Discovery Transcriptomic Group). All patients who were affected by diabetes, systemic autoimmune disorders, infectious diseases, diabetes, chronic lung diseases, neoplasms, or inflammatory diseases, and those receiving antibiotics, corticosteroids, or non-steroidal anti-inflammatory agents were excluded from the study. No patient had symptomatic coronary artery disease or a family history of premature cardiovascular disease.

The study was conducted in accordance with the Declaration of Helsinki and approved by the ethics committee regione Calabria (protocol code 289/2024). The main demographic and clinical characteristics of the patients are summarized in Table 1.

### 4.2. Peripheral Blood Mononuclear Cell (PBMC) Isolation

For all patients included in the study, 15–20 mL samples of whole blood were collected. PBMCs were isolated using density-gradient centrifugation on Ficoll-Paque^TM^ PLUS (Cytiva, Uppsala, Sweden) and SepMate^TM^-50 tubes (STEMCELL Technologies, Vancouver, BC, Canada), according to the manufacturers’ instructions. Cells were counted, and their viability was determined by trypan blue exclusion.

### 4.3. Sample Preparation, Mass Spectrometry Analysis, and Raw Data Processing

The PBMCs were denatured, reduced, and alkylated in 50 µL of LYSE buffer (PreOmics, Planegg/Martinsried Germany cat# PO00032) for 10 min at 95 °C with shaking at 1000 rpm. The protein concentration was determined using the tryptophan assay [42]. A total of 30 µg of protein per sample was digested using the protein aggregation capture (PAC) method, automated on a KingFisher^TM^ Apex robotic platform (Thermo Fisher Scientific, Waltham, MA, USA) in a 96-well format, as previously described [43]. Digestion was performed by adding trypsin and LysC at enzyme-to-protein ratios of 1:50 (*w*/*w*) and 1:100 (*w*/*w*), respectively, followed by mixing and incubation at 37 °C for 2.5 h with gentle agitation. The resulting peptides were purified using the iST protocol [44]. Peptide analysis was carried out on a nano-UHPLC-MS/MS system consisting of an Ultimate 3000 RSLC system coupled to a Q Exactive Plus Orbitrap mass spectrometer (Thermo Fisher Scientific). Chromatographic separation was performed on an EASY-Spray column (75 μm × 25 cm, 2 μm particle size; Thermo Fisher Scientific, ES902) at a flow rate of 250 nL/min using a 70 min gradient: 1 min at 2% buffer B (80% *v*/*v* acetonitrile, 5% *v*/*v* dimethyl sulfoxide, 0.1% *v*/*v* formic acid), ramping to 45% B over 50 min, then to 80% B in 3 min, followed by a 1 min wash at 80% B, and 15 min of re-equilibration at 2% B. Mass spectrometry analysis was performed in data-independent acquisition (DIA) mode. Full MS (MS1) scans were acquired in the Orbitrap at 70,000 resolutions (*m*/*z* range: 375–1500), with a normalized AGC target of 3 × 10^6^ and a maximum injection time of 50 ms. DIA was performed using 19 isolation windows of 34 *m*/*z* with 2 *m*/*z* overlap. MS2 scans were acquired at 35,000 resolutions with a normalized AGC target of 3 × 10^6^, a maximum injection time of 50 ms, and a normalized higher-energy collisional dissociation (HCD) energy of 30%. All spectra were acquired in the profile mode under positive ionization. The raw data were analyzed using Spectronaut version 18 (Biognosys AG) in library-free (directDIA) mode with the default settings. Cleavage rules were set for trypsin/P and LysC. Carbamidomethylation (C) was set as a fixed modification; methionine oxidation (M) and N-terminal acetylation were included as variable modifications. The search was performed against the UniProt Homo sapiens reference proteome. Peptide-spectrum matches and protein groups were filtered at a 1% false discovery rate (FDR). Quantification was performed at the MS2 level, with the precursor filtering set to “Identified (Qvalue)” and the imputation strategy set to “Run-wise Imputing” [45,46,47].

Proteomics reagents were purchased from Thermo Fischer Scientific (Waltham, MA, USA) unless otherwise specified. All other chemicals used were of analytical grade and were obtained from Merck (Darmstadt, Germany). All solutions were prepared with deionized water with a resistivity of not less than 18.2 MΩ cm^−1^.

### 4.4. Analysis of Transcriptome Profile

For the transcriptomic part of the study, we analyzed our previous gene expression microarray data (GeneChip^TM^ Human Genome U133 Array) obtained from PBMCs isolated from 5 CTRs, 10 PD patients and 17 HD patients [15,16].

### 4.5. Measurement of SA β-Galactosidase Activity

The activity of senescence-associated β-galactosidase (SA-β-gal) was assessed using flow cytometry and the CellEvent Senescence Green Flow Cytometry assay kit (Thermo Fisher Scientific, Inchinnan, UK). Freshly isolated PBMCs from 5 CTRs, 5 PD patients, and 5 HD patients were cultured in RPMI-1640 medium (Gibco, Thermo Fisher Scientific, Inchinnan,, UK) supplemented with 2 mM L-glutamine, penicillin (100 U/mL), and streptomycin (100 mg/mL) (Gibco) and treated for 18 h with 100 µM H_2_O_2_. Cells were then washed with PBS and fixed in PBS containing 4% of paraformaldehyde for 10 min at room temperature in the dark. After washing with 1% bovine serum albumin (BSA) in PBS, cells were incubated with the Cell Event^TM^ Senescence Green probe (1:1000) for 2 h at 37 °C in a CO_2_-free incubator protected from light. After incubation, the samples were washed, resuspended in PBS, and analyzed immediately by flow cytometry with the cytoflex (Beckman Coulter, Brea, California, United States). Although a viability assay was not performed, the applied gating strategy allowed an acceptable discrimination of cellular events from debris. A minimum of 10,000 events per sample were acquired and negative controls were included to define background fluorescence.

### 4.6. Western Blot Analysis

To verify antibody specificity, Western blot analysis was performed using the same anti-IRF8 antibody employed for the ELISA (Figure S2). Total proteins from PBMCs were separated by SDS-PAGE on 8–16% gradient gels and transferred onto nitrocellulose membranes (Bio-Rad, Bio-Rad Laboratories, Hercules, CA, USA, Cat# 1620112) using the Trans-Blot SD semi-dry transfer system (Bio-Rad, Cat# 1703940). Membranes were blocked overnight at 4 °C in 3% BSA dissolved in PBS and then washed three times with PBST (PBS with 0.05% Tween-20).

Blots were incubated overnight at 4 °C with a polyclonal rabbit anti-human IRF8 antibody (Merck, Darmstadt, Germany, Cat# HPA002531; 1:1000 in PBST with 3% BSA). After washing, membranes were incubated with an HRP-conjugated goat anti-rabbit IgG secondary antibody (Bio-Rad, Cat# 1706515) for 2 h at room temperature. Following additional washes, signal detection was performed using the SuperSignal™ West Pico PLUS Chemiluminescent Substrate (Thermo Fisher Scientific, Cat# 1863096). The ChemiDoc Imaging System (Bio-Rad, Cat# 12003153) was used to acquire chemiluminescent signals.

### 4.7. Enzyme-Linked Immunosorbent Assay (ELISA) for Interferon Regulatory Factor 8 (IRF8)

IRF8 levels were quantified in the validation cohort using an in-house direct ELISA assay. Briefly, 96-well plates were coated with 5 µg of total proteins from PBMCs diluted in PBS. After incubation, the wells were washed three times with PBST and incubated with 100 µL of polyclonal rabbit anti-human IRF8 antibody (Merck, Cat# HPA002531; dilution 1:1000 in PBST with 3% BSA). After three additional washes with PBST, the wells were incubated with HRP-conjugated goat anti-rabbit IgG (Bio-Rad, Cat# 1706515; dilution 1:10000 in PBST with 1% BSA) for 2 h at room temperature. Plates were then washed again with PBST and developed using 100 µL of TMB substrate solution (Bio-Rad, Cat# 1721066). The enzymatic reaction was stopped by adding 100 µL of 100 mM H_2_SO_4_, and the absorbance was measured at 450 nm using the iMark microplate reader (Bio-Rad). Each sample was analyzed in triplicate, and the mean absorbance value was used for subsequent statistical analyses. The limit of detection was defined as the lowest signal distinguishable from the blank. Absorbance values were corrected by subtracting the background signal and expressed as Relative Units (RU/mL), calculated from a calibration curve generated by serial 1:2 dilutions of the sample exhibiting the highest IRF8 signal, which was used as the reference standard. A positive control, a negative control, and the calibration curve were included in each plate to monitor assay reproducibility and inter-assay variability. In parallel, the wells processed without primary antibody were included as negative controls to assess nonspecific signal.

### 4.8. Statistical Analysis

An appropriate sample size was selected a priori for the validation cohort based on the effect observed in the proteomics discovery phase and according to the equations described by Forshed [48]. Assuming a significance level of (alpha) ≤ 0.05, a statistical power of 80% (1-β = 0.80), an effect size of Cohen’s f = 0.60, and an estimated variance of approximately 0.05, the calculation performed using OriginLab Pro 2025 software indicated a minimum total sample size of approximately 42 subjects.

Statistical analysis was performed using bioinformatic algorithms as previously described [49], adapted for our datasets. Briefly, after log2 transformation, proteins were filtered to retain only those present in at least 70% of the samples within at least one clinical group. Missing values were imputed using a normal distribution, and the entire dataset was normalized using the quantile normalization method. Unsupervised analyses, including principal component analysis (PCA) and hierarchical clustering, were used to assess sample dissimilarities and identify potential outliers.

We applied one-way analysis of variance (ANOVA) to identify proteins showing significant differences across the three groups (CTR, PD, and HD), whereas partial least squares-discriminant analysis (PLS-DA) was used as an exploratory multivariate approach to visualize group separation and rank proteins according to their Variable Importance in Projection (VIP) scores. The first two latent components of the PLS-DA model were used to visualize sample distribution. Additionally, unpaired t-tests were used to identify differentially expressed proteins in pairwise comparisons: CTR vs. PD, CTR vs. HD, and HD vs. PD. For the ANOVA analysis, proteins were considered significantly differentially expressed across the three groups if they met the following criteria: power ≥80% and adjusted *p*-value ≤0.05 after multiple testing correction using the FDR. The results were visualized using a heatmap, where each row represents a protein and each column represents a sample. Protein abundance was normalized with Z-scores and displayed using a pseudocolor scale (red: upregulated, white: mean abundance, blue: downregulated). A hierarchical clustering dendrogram was also included to highlight similarities in protein expression patterns across samples. For the t-test analysis, significance was defined by a statistical power ≥80%, an FDR-adjusted *p*-value ≤0.05, and an absolute log2 fold change ≥1. The results were visualized using volcano plots, in which the significance threshold was graphically represented by the hyperbolic function y = c/(x − x_0_), as previously described [50]. The same statistical filtering was applied to the transcriptomic analysis.

Functional enrichment analysis was performed on the combined list of statistically significant transcripts and proteins identified in each pairwise comparison using the PathfindR package for R software version 4.5.3 [51]. This has led to the identification of significantly enriched biological processes and pathways based on KEGG and IntAct annotations with 25 iterations. For visualization purposes, the enriched terms identified were subsequently grouped into seven major functional categories and displayed as a two-dimensional bubble plot. Kinase enrichment analysis (KEA) was performed using the combined set of statistically significant transcripts and proteins identified in the HD vs. PD comparison to prioritize candidate upstream kinases potentially regulating the observed molecular signature. The analysis integrates data from 11 curated kinase–substrate and protein–protein interactions databases to generate an integrated ranking of candidate upstream kinases. Kinases were ranked based on their integrated MeanRank score, with lower Sum of Ranks values indicating stronger predicted kinase enrichment [52]. The identified kinases were visualized using the Coral web application [53].

For the ELISA validation of IRF8 protein levels in PBMCs, the Kruskal–Wallis test was used to assess differences among groups. Moreover, pairwise comparisons were performed using Dunn’s test with Benjamini–Hochberg adjustment for multiple testing. Results are expressed as medians with interquartile ranges (IQRs). All statistical analyses were conducted using OriginLab Pro 2025 and R version 4.5.3.

## Figures and Tables

**Table 1:** Demographic and clinical characteristics of the patient population.

	Discovery Group				Validation Group			
	HD	PD	CTR	*p* Value	HD	PD	CTR	*p* Value
Number	5	5	5	/	22	16	10	/
Gender (M/F)	2/3	3/2	3/2	/	10/12	8/8	6/4	/
Age (years)	49.2 ± 7.9	56.4 ± 10.0	45.4 ± 9.2	0.193	51.2 ± 5.9	48.9 ± 3.6	49.1 ± 6.7	0.376
Time on dialysis (years)	4.9 ± 3.5	3.8 ± 4.5	/	0.677	4.5 ± 2.8	3.6 ± 5.4	/	0.507
BMI (kg/m^2^)	23.8 ± 4.6	24.7 ± 3.0	22.2 ± 3.7	0.592	22.5 ± 2.1	23.7 ± 3.2	22.3 ± 2.2	0.272
Systolic blood pressure (mmHg)	128.8 ± 22.0	138 ± 13.0	118 ± 13.4	0.206	126.4 ± 21.1	130 ± 13.0	117 ± 21.0	0.232
Diastolic blood pressure (mmHg)	84.2 ± 9.9	86.6 ± 9.8	78.4 ± 5.4	0.336	86.4 ± 8.6	87.3 ± 9.0	80.6 ± 6.1	0.118
Total protein (g/dL)	6.4 ± 0.7	6.1 ± 0.4	6.6 ± 2.4	0.864	7.0 ± 1.1	6.2 ± 1.6	6.4 ± 0.8	0.135
Hemoglobin (g/dL)	11.5 ± 1.7	11.8 ± 0.7	12.6 ± 0.6	0.309	11.2 ± 1.1	11.3 ± 0.7	12.0 ± 1.2	0.114

*p* value by ANOVA; time on dialysis by *t*-test.

## Data Availability

The mass spectrometry proteomics data have been deposited to the ProteomeXchange Consortium via the PRIDE partner repository (https://www.ebi.ac.uk/pride/profile/reviewer_pxd076533) with the following dataset identifier: PXD076533. Results of the microarray experiment are available in the Gene Expression Omnibus (GSE15072).

## Supplementary Materials

The following supporting information can be downloaded at https://www.mdpi.com/article/10.3390/ijms27167470/s1.

## Abbreviations

The following abbreviations are used in this manuscript:

CKD	Chronic kidney disease
HD	Hemodialysis
PD	Peritoneal dialysis
PBMCs	Peripheral blood mononuclear cells
CTR	Healthy control
PLS-DA	Partial least squares-discriminant analysis
VIP	Variable Importance in Projection
IRF8	Interferon regulatory factor 8
GO	Gene Ontology
CHEK2	Checkpoint Kinase 2
SA-β-gal	Senescence-Associated β-Galactosidase
SASP	Senescence-associated secretory phenotype
HCD	Higher-energy collisional dissociation
BSA	Bovine serum albumin
ELISA	Enzyme-linked immunosorbent assay
ANOVA	Analysis of variance
KEA	Kinase enrichment analysis
IQR	Interquartile range
