# Supplementary material for: Multi-Omics Analysis of PBMCs Revealed Distinct Biological Differences Between Hemodialysis and Peritoneal Dialysis
Source: Int J Mol Sci. 2026 Aug 20;27(16):7470. doi: 10.3390/ijms27167470 (PMC13512927; doi:10.3390/ijms27167470)
Supplement: Supplementary file 1 [file ijms-27-07470-s001.zip › ijms-4436702-supplementary.pdf]

Supplementary Material

Supplementary Figures

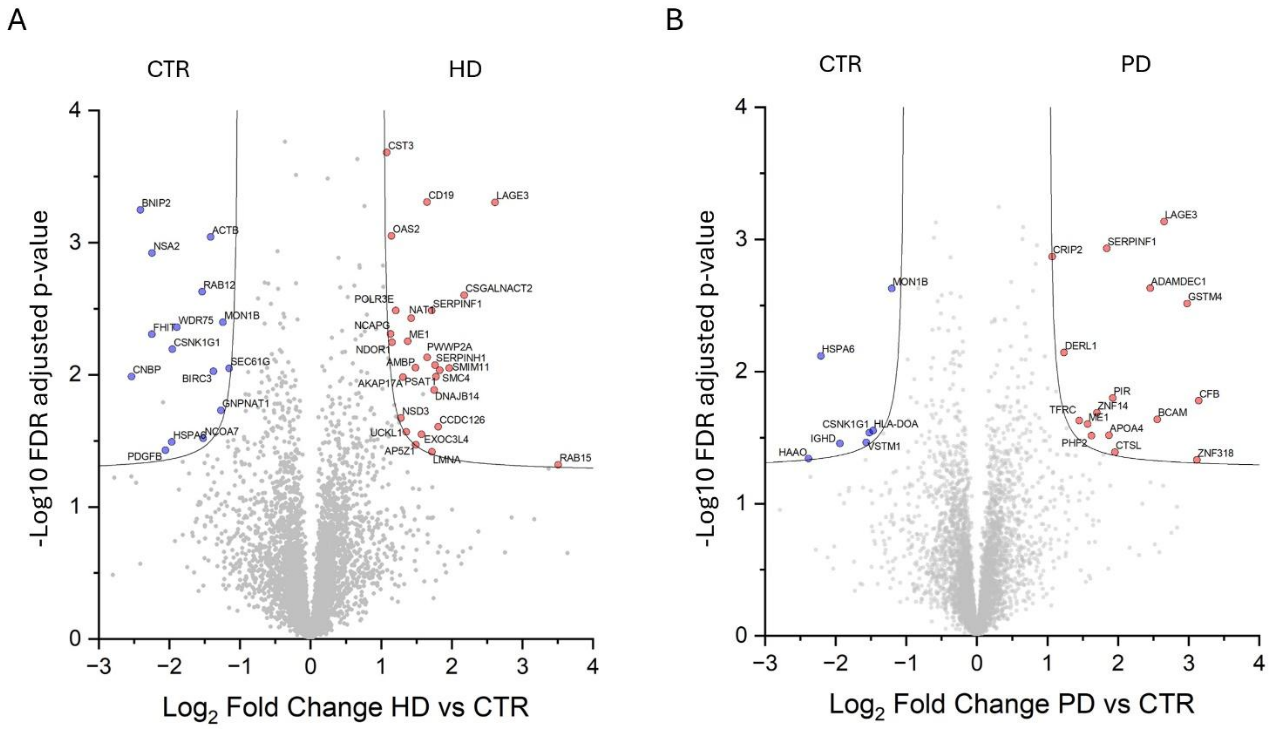

Figure S1. Volcano plots of differentially abundant proteins in the comparison (A) CTR vs HD and (B) CTR vs PD. Red and blue dots indicate proteins that were more or less abundant in HD and PD compared with CTR, respectively, whereas gray dots indicate proteins without statistically significant differences. Black lines indicate the significance thresholds (|log2 fold change| ≥ 1 and FDR-adjusted p-value ≤ 0.05), as defined by the hyperbolic threshold applied in the differential expression analysis.

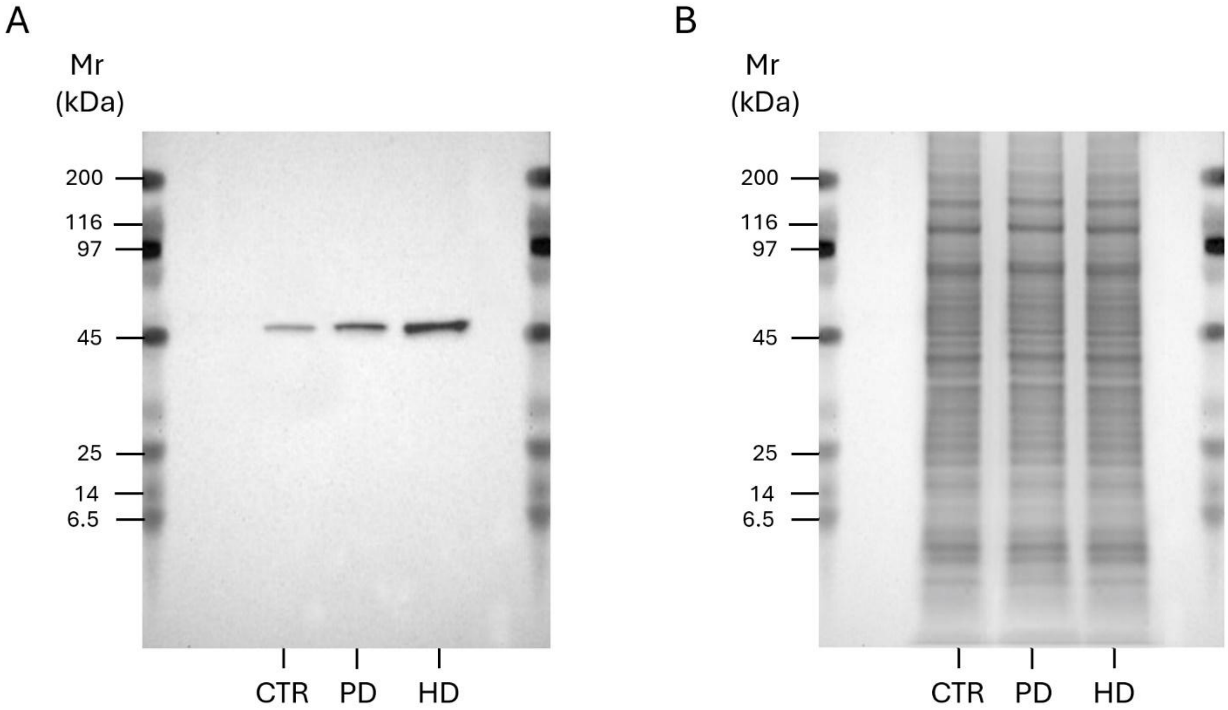

Figure S2. Western blot validation of the anti-IRF8 antibody specificity. (A) Representative Western blot showing IRF8 immunoreactivity in total proteins from PBMCs of CTR, PD, and HD. A single immunoreactive band was detected at the expected molecular weight (~48 kDa) of IRF8, supporting the specificity of the anti-IRF8 antibody used for ELISA validation. **(B)** Corresponding Ponceau S staining of the same membrane. Total lane protein staining was used as the loading control and for normalization of IRF8 band intensity.

Supplementary tables

Table S1. Differentially abundant proteins identified by one-way ANOVA and pairwise comparisons among CTR, PD, and HD groups. The table reports the proteins identified as significantly different among the three study groups by one-way ANOVA. For each protein, the gene symbol, protein name, log2 fold change (log2FC), and −log10 FDR adjusted p-value are reported for the pairwise comparisons CTR vs HD, CTR vs PD, and PD vs HD.

Gene symbol	Protein name	Log2 FC PD vs CTR	-Log10 FDR adj. p-value PD vs CTR	Log2 FC HD vs CTR	-Log10 FDR adj. p-value HD vs CTR	Log2 FC HD vs PD	-Log10 FDR adj. p-value HD vs PD
PHF2	Lysine-specific demethylase PHF2	1.6231	1.5154	0.1968	0.0780	-1.4263	1.5292
DNAJB14	DnaJ homolog subfamily B member 14	0.9412	0.9485	1.7493	1.8857	0.8081	1.0828
BCAM	Basal cell adhesion molecule	2.5535	1.6391	1.3290	0.4140	-1.2246	0.6347
SYNJ2BP-COX16	Synaptojanin-2-binding protein	0.5122	0.4136	-0.8200	0.5254	-1.3322	1.8739
OAS2	2'-5'-oligoadenylate synthase 2	0.8506	2.9557	1.1437	3.0511	0.2931	0.9897
CHEK2	Serine/threonine-protein kinase Chk2	-1.3491	0.5181	1.2778	0.6080	2.6269	1.6704
IGHD	Immunoglobulin heavy constant delta	-1.9395	1.4563	0.3953	0.3006	2.3347	1.5880
SCOC	Short coiled-coil protein	0.7853	1.9850	1.2052	2.4869	0.4199	1.0756
POLR3E	DNA-directed RNA polymerase III subunit RPC5	-1.5218	1.5382	-1.9577	2.1946	-0.4358	0.2876
CSNK1G1	Casein kinase I isoform gamma-1	0.4111	0.6425	-1.2229	0.9372	-1.6341	1.6244
HLA-DRB3	HLA class II histocompatibility antigen, DR beta 3 chain	-1.5667	1.4643	-0.3072	0.4695	1.2594	1.1701
HNRNPU	Heterogeneous nuclear ribonucleoprotein U	0.0481	0.0553	-1.8648	1.1293	-1.9129	1.3988
CBLB	E3 ubiquitin-protein ligase CBL-B	0.1054	0.1012	-1.4171	3.0435	-1.5225	2.5336
VSTM1	V-set and transmembrane domain-containing protein 1	2.6388	1.1363	3.5077	1.3194	0.8688	0.2263
GIPC3	PDZ domain-containing protein GIPC3	0.3594	0.1882	1.4911	1.4718	1.1317	0.8289
ACTB	Actin, cytoplasmic 1	-0.2258	0.1127	-1.5370	2.6298	-1.3113	0.8771
RAB15	Ras-related protein Rab-15	0.3263	0.2953	-1.0237	1.4251	-1.3500	1.8620
RAB12	Ras-related protein Rab-12	-0.8311	1.1796	-1.3759	2.0271	-0.5448	0.4961
SERPING1	Plasma protease C1 inhibitor	1.9559	1.3909	1.4556	0.7830	-0.5003	0.6475
CFB	Complement factor B	1.4492	1.6296	1.6578	0.7917	0.2086	0.0740
TSSC4	U5 small nuclear ribonucleoprotein TSSC4	3.1406	1.7804	1.8369	0.7007	-1.3037	0.5382
DERL1	Derlin-1	-0.4352	0.4338	0.7570	0.9088	1.1922	2.2145
ANKZF1	tRNA endonuclease ANKZF1	-0.0972	0.0727	-1.3806	1.3001	-1.2834	1.6549
HAAO	3-hydroxyanthranilate 3,4-dioxygenase	1.2329	2.1449	0.8925	1.3143	-0.3404	0.5384
CCDC126	Coiled-coil domain-containing protein 126	1.5955	1.1809	-0.4446	0.1782	-2.0402	2.6311
OXNAD1	Oxidoreductase NAD-binding domain-containing protein 1	-2.3833	1.3421	-1.6734	0.8035	0.7099	0.2559
RFESD	Rieske domain-containing protein	1.2014	1.1287	1.8080	1.6076	0.6066	1.4060
SMC4	Structural maintenance of chromosomes protein 4	-0.8252	2.6301	0.3160	0.7717	1.1413	2.9018
SERPINH1	Serpin H1	-0.6367	0.1099	3.1701	0.9068	3.8068	1.3585
TRRAP	Transformation/transcription domain-associated protein	0.0874	0.0941	1.2546	1.6371	1.1672	2.1462
NAT1	Arylamine N-acetyltransferase 1	-1.2046	2.6291	-1.2429	2.3989	-0.0383	0.0343
POLDIP3	Polymerase delta-interacting protein 3	0.4051	0.1559	1.8283	2.0351	1.4232	0.6971

TXNDC15	Thioredoxin domain-containing protein 15	-0.3419	0.2727	1.7627	2.0730	2.1046	3.2876
MRPL11	Large ribosomal subunit protein uL11m	-0.5023	0.8701	0.7597	0.9013	1.2620	2.7534
IRF8	Interferon regulatory factor 8	0.5873	0.7626	1.4244	2.4285	0.8371	1.0396
BNIP2	BCL2/adenovirus E1B 19 kDa protein-interacting protein 2	-0.5792	0.6696	-1.2701	1.7322	-0.6909	0.6039
FEZ2	Fasciculation and elongation protein zeta-2	0.5467	1.0507	1.2777	1.6744	0.7310	1.2356
CCNYL1	Cyclin-Y-like protein 1	1.0666	2.8709	-0.0883	0.0716	-1.1549	1.8975
STRN4	Striatin-4	-0.7179	0.8337	0.5726	0.6956	1.2905	2.4821
PIR	Pirin	-0.7808	1.2371	-2.4108	3.2480	-1.6299	2.7364
TNFRSF10C	Tumor necrosis factor receptor superfamily member 10C	-1.2873	1.1579	0.3313	0.4539	1.6187	1.5101
ADAMDEC1	ADAM DEC1	1.9243	1.8008	1.4775	0.9043	-0.4467	0.2857
NSA2	Ribosome biogenesis protein NSA2 homolog	-0.2377	0.1243	1.5293	0.9966	1.7670	1.4470
PDGFB	Platelet-derived growth factor subunit B	-0.3275	0.2928	0.8737	0.9179	1.2012	2.2911
AMBP	Protein AMBP	2.4544	2.6321	0.9450	0.4037	-1.5093	0.9725
HLA-DOA	HLA class II histocompatibility antigen, DO alpha chain	-0.6868	0.4689	-2.2473	2.9221	-1.5605	1.1082
APOA4	Apolipoprotein A-IV	0.7004	2.2661	1.0775	3.6829	0.3770	1.2652
CA3	Carbonic anhydrase 3	-0.6218	0.8105	-2.0551	1.4302	-1.4333	1.0856
GSTM1	Glutathione S-transferase Mu 1	0.7762	0.7504	1.7180	1.4187	0.9418	0.6361
TUBA3C	Tubulin alpha-3C chain	0.8695	1.1612	1.4848	2.0545	0.6153	0.7783
COX6A1	Cytochrome c oxidase subunit 6A1, mitochondrial	-1.4693	1.5536	-0.1486	0.1226	1.3208	1.1880
CD19	B-lymphocyte antigen CD19	1.8706	1.5185	1.4112	0.8193	-0.4594	0.3359
HSPA6	Heat shock 70 kDa protein 6	1.0806	1.2390	-1.4427	1.0650	-2.5233	2.6782
CD33	Myeloid cell surface antigen CD33	0.7358	0.3357	3.6061	4.2328	2.8703	2.0575
GSTM3	Glutathione S-transferase Mu 3	0.3283	0.3412	-1.3491	0.9611	-1.6774	1.4470
GRK3	G protein-coupled receptor kinase 3	0.0603	0.0508	1.6485	3.3069	1.5882	2.4786
SERPINF1	Pigment epithelium-derived factor	1.6995	1.6893	1.0799	0.8664	-0.6196	0.7473
ME1	NADP-dependent malic enzyme	-2.2091	2.1192	-1.9677	1.4925	0.2414	0.0993
FHIT	Bis(5'-adenosyl)-triphosphatase	0.3450	0.4668	-1.0543	1.2728	-1.3993	2.1602
SMIM11	Small integral membrane protein 11	1.8378	2.9326	1.7146	2.4866	-0.1232	0.1719
CNBP	CCHC-type zinc finger nucleic acid binding protein	1.5683	1.6037	1.3761	2.2551	-0.1922	0.1310
AKAP17A	A-kinase anchor protein 17A	-1.6106	1.1233	-2.2483	2.3082	-0.6377	0.2880
GSTM4	Glutathione S-transferase Mu 4	1.3306	1.2009	1.9634	2.0521	0.6328	0.7302
ANK3	Ankyrin-3	-0.5252	1.2473	-1.1551	2.0501	-0.6298	0.9468
EIF4EBP1	Eukaryotic translation initiation factor 4E-binding protein 1	-1.3260	1.5328	-2.5355	1.9870	-1.2095	0.7526
LAGE3	EKC/KEOPS complex subunit LAGE3	0.3875	0.4972	1.3069	1.9830	0.9194	2.3640
PWP2	Periodic tryptophan protein 2 homolog	2.9777	2.5144	0.6873	0.5344	-2.2904	1.6705
HMGN3	High mobility group nucleosome-binding domain-containing protein 3	0.2943	0.2008	-1.4644	0.9256	-1.7587	1.7227
FSCN1	Fascin-1	2.6518	3.1354	2.6105	3.3050	-0.0413	0.0327
EIF2S3B	Eukaryotic translation initiation factor 2 subunit 3B	1.5023	1.2170	-1.2256	0.5851	-2.7278	2.2382
OCIAD2	OCIA domain-containing protein 2	-0.0538	0.0481	1.1859	1.7713	1.2396	2.9219
ZNF318	Zinc finger protein 318	0.3544	0.1986	1.5705	1.5508	1.2160	0.8285
LYPD2	Ly6/PLAUR domain-containing protein 2	3.1150	1.3317	2.1557	0.6624	-0.9593	0.9339
WDR75	WD repeat-containing protein 75	-0.0310	0.0576	-1.3496	1.5435	-1.3186	1.8880
PTGR3	Prostaglandin reductase 3	0.1264	0.3409	-1.8946	2.3613	-2.0211	2.7886
CSGALNACT2	Chondroitin sulfate N-acetylgalactosaminyltransferase 2	0.1453	0.2729	-1.2543	1.2599	-1.3996	1.6583

NCOA7	Nuclear receptor coactivator 7	0.7065	0.7128	2.1760	2.6034	1.4695	2.0689
PRCC	Proline-rich protein PRCC	-0.4887	0.3676	1.4486	1.4703	1.9373	1.7083
SH3KBP1	SH3KBP1-binding protein 1	-0.5134	1.3719	-1.5227	1.5204	-1.0092	1.0347
RNF25	E3 ubiquitin-protein ligase RNF25	1.0188	0.8311	-0.6980	0.3046	-1.7169	1.6071
PWWP2A	PWWP domain-containing protein 2A	1.2987	0.8767	1.6512	2.1323	0.3525	0.1850
ALG2	Alpha-1,3/1,6-mannosyltransferase ALG2	0.2233	0.4281	1.1315	2.3103	0.9082	2.2606
MEAF6	Chromatin modification-related protein MEAF6	0.5143	0.7546	1.3548	1.5692	0.8405	1.5282
IRAK4	Interleukin-1 receptor-associated kinase 4	0.1867	0.2241	1.1531	2.2464	0.9663	1.2704
STK38L	Serine/threonine-protein kinase 38-like	-0.0893	0.0985	-1.3191	1.3578	-1.2299	1.7042
PSAT1	Phosphoserine aminotransferase	0.6619	1.0334	1.7763	1.9869	1.1144	1.7943

Table S2. List of proteins discriminating between HD and PD. The table reports the 36 proteins that were significantly differentially abundant between HD and PD. Gene symbol, protein name, log2 fold change (log2FC), and −log10 FDR adjusted p-value are reported.

	**Gene name**	**Protein name**	**Log2 Fold Change HD vs PD**	**-Log10 FDR adj p-value HD vs PD**
**1**	TUBA3C	Tubulin alpha-3C chain	2.8703	2.0575
**2**	CHEK2	Serine/threonine-protein kinase Chk2	2.6269	1.6704
**3**	IGHD	Immunoglobulin heavy constant delta	2.3347	1.5880
**4**	SERPINH1	Serpin H1	2.1046	3.2876
**5**	GALNT6	Polypeptide N-acetylgalactosaminyltransferase 6	1.9373	1.7083
**6**	TRDMT1	tRNA (cytosine(38)-C(5))-methyltransferase	1.7670	1.4470
**7**	STRN4	Striatin-4	1.6187	1.5101
**8**	CD19	B-lymphocyte antigen CD19	1.5882	2.4786
**9**	CSGALNACT2	Chondroitin sulfate N-acetylgalactosaminyltransferase 2	1.4695	2.0689
**10**	IRF8	Interferon regulatory factor 8	1.2905	2.4821
**11**	TRRAP	Transformation/transcription domain-associated protein	1.2620	2.7534
**12**	FSCN1	Fascin-1	1.2396	2.9219
**13**	TNFRSF10C	Tumor necrosis factor receptor superfamily member 10C	1.2012	2.2911
**14**	FCGR3A	Low affinity immunoglobulin gamma Fc region receptor III-A	1.1922	2.2145
**15**	SLC36A1	Proton-coupled amino acid transporter 1	1.1672	2.1462
**16**	OXNAD1	Oxidoreductase NAD-binding domain-containing protein 1	1.1413	2.9018
**17**	NENF	Neudesin	-1.2299	1.7042
**18**	TSSC4	U5 small nuclear ribonucleoprotein TSSC4	-1.2834	1.6549
**19**	SDE2	Splicing regulator SDE2	-1.3186	1.8880
**20**	SYNJ2BP-COX16	Synaptojanin-2-binding protein	-1.3322	1.8739
**21**	KTI12	Protein KTI12 homolog	-1.3500	1.8620
**22**	GSTM3	Glutathione S-transferase Mu 3	-1.3993	2.1602
**23**	PTGR3	Prostaglandin reductase 3	-1.3996	1.6583
**24**	PHF2	Lysine-specific demethylase PHF2	-1.4263	1.5292
**25**	ACTB	Actin, cytoplasmic 1	-1.5225	2.5336
**26**	BNIP2	BCL2/adenovirus E1B 19 kDa protein-interacting protein	-1.6299	2.7364
**27**	HNRNPU	Heterogeneous nuclear ribonucleoprotein U	-1.6341	1.6244
**28**	COX6A1	Cytochrome c oxidase subunit 6A1, mitochondrial	-1.6774	1.4470
**29**	SH3KBP1	SH3KBP1-binding protein 1	-1.7169	1.6071
**30**	ANK3	Ankyrin-3	-1.7587	1.7227
**31**	GIPC3	PDZ domain-containing protein GIPC3	-1.9129	1.3988
**32**	WDR75	WD repeat-containing protein 75	-2.0211	2.7886
**33**	ANKZF1	tRNA endonuclease ANKZF1	-2.0402	2.6311
**34**	GSTM4	Glutathione S-transferase Mu 4	-2.2904	1.6705
**35**	GSTM1	Glutathione S-transferase Mu 1	-2.5233	2.6782

**36**	HMGN3	High mobility group nucleosome-binding domain-containing protein 3	-2.7278	2.2382

Table S3. List of proteins discriminating between HD and CTR. The table reports the 41 proteins that were significantly differentially abundant between HD and CTR. Gene symbol, protein name, log2 fold change (log2FC), and −log10 FDR adjusted p-value are reported.

	**Gene name**	**Protein name**	**Log2 Fold Change HD vs CTR**	**-Log10 FDR adj p-value HD vs CTR**
**1**	RAB15	Ras-related protein Rab-15	3.5077	1.3194
**2**	LAGE3	EKC/KEOPS complex subunit LAGE3	2.6105	3.3050
**3**	CSGALNACT2	Chondroitin sulfate N-acetylgalactosaminyltransferase 2	2.1760	2.6034
**4**	SMIM11	Small integral membrane protein 11	1.9634	2.0521
**5**	SMC4	Structural maintenance of chromosomes protein 4	1.8283	2.0351
**6**	CCDC126	Coiled-coil domain-containing protein 126	1.8080	1.6076
**7**	PSAT1	Phosphoserine aminotransferase	1.7763	1.9869
**8**	SERPINH1	Serpin H1	1.7627	2.0730
**9**	DNAJB14	DnaJ homolog subfamily B member 14	1.7493	1.8857
**10**	LMNA	Prelamin-A/C	1.7180	1.4187
**11**	SERPINF1	Pigment epithelium-derived factor	1.7146	2.4866
**12**	PWWP2A	PWWP domain-containing protein 2A	1.6512	2.1323
**13**	CD19	B-lymphocyte antigen CD19	1.6485	3.3069
**14**	EXOC3L4	Exocyst complex component 3-like protein 4	1.5705	1.5508
**15**	AP5Z1	AP-5 complex subunit zeta-1	1.4911	1.4718
**16**	AMBP	Protein AMBP	1.4848	2.0545
**17**	NAT1	Arylamine N-acetyltransferase 1	1.4244	2.4285
**18**	ME1	NADP-dependent malic enzyme	1.3761	2.2551
**19**	UCKL1	Uridine-cytidine kinase-like 1	1.3548	1.5692
**20**	AKAP17A	A-kinase anchor protein 17A	1.3069	1.9830
**21**	NSD3	Histone-lysine N-methyltransferase NSD3	1.2777	1.6744
**22**	POLR3E	DNA-directed RNA polymerase III subunit RPC5	1.2052	2.4869
**23**	NDOR1	NADPH-dependent diflavin oxidoreductase 1	1.1531	2.2464
**24**	OAS2	2'-5'-oligoadenylate synthase 2	1.1437	3.0511
**25**	NCAPG	Condensin complex subunit 3	1.1315	2.3103
**26**	CST3	Cystatin-C	1.0775	3.6829
**27**	SEC61G	Protein transport protein Sec61 subunit gamma	-1.1551	2.0501
**28**	MON1B	Vacuolar fusion protein MON1 homolog B	-1.2429	2.3989
**29**	GNPNAT1	Glucosamine 6-phosphate N-acetyltransferase	-1.2701	1.7322
**30**	BIRC3	Baculoviral IAP repeat-containing protein 3	-1.3759	2.0271
**31**	ACTB	Actin, cytoplasmic 1	-1.4171	3.0435
**32**	NCOA7	Nuclear receptor coactivator 7	-1.5227	1.5204
**33**	RAB12	Ras-related protein Rab-12	-1.5370	2.6298
**34**	WDR75	WD repeat-containing protein 75	-1.8946	2.3613
**35**	CSNK1G1	Casein kinase I isoform gamma-1	-1.9577	2.1946
**36**	HSPA6	Heat shock 70 kDa protein 6	-1.9677	1.4925
**37**	PDGFB	Platelet-derived growth factor subunit B	-2.0551	1.4302
**38**	NSA2	Ribosome biogenesis protein NSA2 homolog	-2.2473	2.9221
**39**	FHIT	Bis(5'-adenosyl)-triphosphatase	-2.2483	2.3082
**40**	BNIP2	BCL2/adenovirus E1B 19 kDa protein-interacting protein 2	-2.4108	3.2480
**41**	CNBP	CCHC-type zinc finger nucleic acid binding protein	-2.5355	1.9870

Table S4. List of proteins discriminating between PD and CTR. The table reports the 23 proteins that were significantly differentially abundant between PD and CTR. Gene symbol, protein name, log2 fold change (log2FC), and −log10 FDR adjusted p-value are reported.

	Gene name	Protein name	Log2 Fold Change PD vs CTR	-Log10 p-value PD vs CTR
**1**	CFB	Complement factor B	3.1406	1.7804
**2**	ZNF318	Zinc finger protein 318	3.1150	1.3317
**3**	GSTM4	Glutathione S-transferase Mu 4	2.9777	2.5144
**4**	LAGE3	EKC/KEOPS complex subunit LAGE3	2.6518	3.1354
**5**	BCAM	Basal cell adhesion molecule	2.5535	1.6391
**6**	ADAMDEC1	ADAM DEC1	2.4544	2.6321
**7**	CTSL	CTD small phosphatase-like protein 2	1.9559	1.3909
**8**	PIR	Pirin	1.9243	1.8008
**9**	APOA4	Apolipoprotein A-IV	1.8706	1.5185
**10**	SERPINF1	Pigment epithelium-derived factor	1.8378	2.9326
**11**	ZNF14	Zinc finger protein 14	1.6995	1.6893
**12**	PHF2	Lysine-specific demethylase PHF2	1.6231	1.5154
**13**	ME1	NADP-dependent malic enzyme	1.5683	1.6037
**14**	TFRC	Transferrin receptor protein 1	1.4492	1.6296
**15**	DERL1	Derlin-1	1.2329	2.1449
**16**	CRIP2	Cysteine-rich protein 2	1.0666	2.8709
**17**	MON1B	Vacuolar fusion protein MON1 homolog B	-1.2046	2.6291
**18**	HLA-DOA	HLA class II histocompatibility antigen, DO alpha chain	-1.4693	1.5536
**19**	CSNK1G1	Casein kinase I isoform gamma-1	-1.5218	1.5382
**20**	VSTM1	V-set and transmembrane domain-containing protein 1	-1.5667	1.4643
**21**	IGHD	Immunoglobulin heavy constant delta	-1.9395	1.4563
**22**	HSPA6	Heat shock 70 kDa protein 6	-2.2091	2.1192
**23**	HAAO	3-hydroxyanthranilate 3,4-dioxygenase	-2.3833	1.3421

Table S5. Functional enrichment analysis performed using pathfindR on the combined set of differentially expressed proteins and transcripts. Functional enrichment analysis was performed using pathfindR on the combined set of significantly differentially expressed proteins and transcripts. Enriched functional terms and pathways are reported for the PD vs CTR, HD vs CTR, and HD vs PD comparisons. Fold enrichment and −log10 FDR adjusted p-values are shown for each comparison. Associated genes indicate the differentially expressed molecules contributing to each enriched term, whereas gene set size represents the total number of genes annotated to the corresponding functional term or pathway

Biological procfesses / Pathways	Functional Group	PD vs CTR Fold Enrichment	PD vs CTR -log10 FDR adj p-value	HD vs CTR Fold Enrichment	HD vs CTR -log10 FDR adj p-value	PD vs HD Fold Enrichment	PD vs HD -log10 FDR adj p-value	Genes associate	Total Size
Apoptosis	Cell fate regulation	3.812910838	5.370166012	6.94494474	6.20868972	3.992117647	1.572015759	BIRC6, AKT2, CFLAR, CASP2, BIRC3, FADD, ENDOG, PDPK1, PRF1, ACTG1, APAF1	11
Cellular senescence	Cell fate regulation	1.835310829	5.309021681	4.156095337	8.124475524	3.084818182	3.226296075	IRF8, BRIC6, BRIC3, CFLAR, AKT2, PDPK1, RPTOR, BCL2, CHEk2	9
Longevity regulating pathway - Aging	Cell fate regulation	0.601082458	3.449854427	3.601082458	3.449854427	2.601082458	3.449854427	AKT2, HSPA8, RPTOR	3
Cell adhesion molecules	Cell structure & transport	0.894291588	1.544529481	3.45703916	2.285273956	2.417618321	3.611136844	CD6, CD2, ITGB7, CD58, HLA-DRA, HLA-DRB1, HLA-B	7
Endocytosis	Cell structure & transport	2.257985094	1.45798171	3.946340193	2.277492166	3.27558371	1.570799262	STAMBP, TFRC, ARAP1, EHD4, RAB8A, ACTR2, SH3KBP1, DNM2, AP2S1, AP2B1, SNF8, LDLRAP1, ARRB2, IQSEC2, HLA-B, ARF4, KIF5B, RAB31, VPS29, VPS35	20
Regulation of actin cytoskeleton	Cell structure & transport	0.780930682	2.031462025	2.700811843	3.739156836	1.892539686	1.506966295	DES, FLNC, NCKAP1L, BAIAP2, DIAPH2, VAV3, AKT2, PIP4K2A, PIP4K2B, PDGFB, ACTG1	11
Glycosaminoglycan biosynthesis - degradation	Metabolism & Biosynthesis	13.13196594	4.562282073	6.173284213	2.118956834	7.232097187	1.666898499	IDUA, GALNS, NAGLU, CSGALNACT2, XYLT2, NDST2	6
Metabolism	Metabolism & Biosynthesis	10.55705882	6.575311333	3.45703916	2.285273956	13.30705882	6.575311333	HK2, HK3, UGP2, UGDH, GPI, GMPPB, GMPPA, PGM3, NANS, NAGK	10
Oxidative phosphorylation	Metabolism & Biosynthesis	1.084818182	2.662045058	5.757393339	7.361409474	4.980990826	2.929045043	NDUFB9, NDUFB8, NDUFV2, NDUFS3, COX6A1, COX2, PPA2, ATP6V1H, ATP6V1A, ATP6V1C1, ATP6V1E1, ATP6AP1	12
Ribosome	Metabolism & Biosynthesis	8.544772361	9.692946067	2.342872924	2.72991055	3.815759036	2.252599042	FAU, RPS29, RPS13, RPS8, RPS10, RPS25, RPS3A, RPL23, RPL17, RPL26, RPL32, RPL27, RPL38, RPL4, RPS5, WDR75,	22

								CSNK2A1, NOP56, TCOF1, FBL, GTPBP4, RAN	
Lysosome	Degradation & Recycling	4.584124595	4.411459083	2.33583727	2.503319233	3.562519685	1.547690231	CTSL, IDUA, PPT1, NAGLU, GALNS, AP1S2, AP1M1, SGSH, LIPA, ATP6V1H, ATP6AP1, CD63, NPC2, SORT1, AP1B1, GGA3	16
Autophagy	Degradation & Recycling	4.167210526	9.547188328	3.45703916	2.285273956	3.921146667	1.712718633	AKT2, MAP3K7, CFLAR, MAP1LC3B2, VPS39, BIRC6, PDPK1, RPTOR, PRKCQ, SQSTM1, PRKCD, PPP2CA, ATG2B, PIK3R4, ATG7, GABARAP, CALCOCO2, VPS33A, STX7, IRF8	20
Phagosome	Degradation & Recycling	3.035369257	2.076149727	6.537226277	4.031998968	4.953722628	3.533285285	CTSL, TFRC, ATP6V1C1, SEC61B, HLA-DOA, ACTB, DYNC1LI2, FCGR3A, NCF4, NCF1, NCF2, ATP6V1H, ATP6V1A, ATP6V1E1, ATP6AP1, STX7, HLA-DRA, HLA-DRB1, HLA-B, IRF8	20
Chemokine signaling pathway	Immune system	1.911933739	4.115126394	1.490103086	1.50804514	2.340206897	3.44955975	LYN, PLCB2, PTK2B, PIK3CG, JAK2, VAV3, AKT2, PXN, RAC2, FGR, HCK, PLCG2, ARRB2, PRKCD, NCF1, PREX1, IRF8	17
Natural killer cell mediated cytotoxicity	Immune system	1.49854266	2.677854064	3.668432432	6.418839495	2.33583727	2.503319233	PTK2B, PTPN11, VAV3, PRF1, LCK, NFATC2, CD48, RAC2, ACTB, PLCG2, SYK, PTPN6, HLA-B, FCGR3A, GSDMD, HAT1, NCF1, NCF2, NCF4, ATG7, SIGLEC9, MAPK14, IRF8	23
T cell receptor signaling pathway	Immune system	1.790541176	7.301355293	4.548735736	4.380281962	3.96877193	6.351250152	AKT2, VAV3, MAP3K7, DLG1, CARD11, PDPK1, NCK1, LCK, PRKCQ, NFATC2, GRAP2, CD3D, CD3G, PTPN6, MAPK14, PPP2CA, IRF8	17
Th1 and Th2 cell differentiation	Immune system	6.544772361	3.136240884	2.287746503	2.036791063	4.790541176	2.325071501	JAK2, LCK, STAT4, PRKCQ, NFATC2, CD3D, CD3G, MAPK14, HLA-DRA, HLA-DRB1	10
C-type lectin receptor signaling pathway	Immune system	1.788583175	2.909384816	4.167210526	9.547188328	3.405462366	1.919753974	CYLD, PTPN11, LSP1, NFATC2, SYK, CARD9, PRKCD, PLCG2, MAPK14, IRF8	10
B cell receptor signaling pathway	Immune system	NaN	NaN	NaN	NaN	4.672105263	9.527699056	NFATC2, RAC2, CD19, INPPL1, PLCG2, SYK, PTPN6, IRF8	8
AMPK signaling pathway	Signal transduction	NaN	NaN	2.893726975	2.491614325	NaN	NaN	AKT2, FASN, MAP3K7, PDPK1, RPTOR	5

JAK-STAT signaling pathway	Signal transduction	NaN	NaN	1.620487106	1.616175498	NaN	NaN	JAK2, AKT2, STAT4, PDGFB, IRF8	5
MAPK signaling pathway	Signal transduction	NaN	NaN	1.473170096	1.790691013	NaN	NaN	PLA2G4A, AKT2, MAP3K7, HSPA8, STK4, PDGFB, IRF8	7
ErbB signaling pathway	Signal transduction	2.428295406	4.532264004	1.892539686	1.506966295	0.990744526	2.460836325	PTPN11, PLCB2, PIK3CG, PTK2B, AKT2, PLA2G4A, PDGFB, PLCG2, DNM2, SYK	10
NF-kappa B signaling pathway	Signal transduction	2.835310829	5.309021681	5.156095337	8.124475524	3.084818182	3.226296075	BCL2, CYLD, LYN, MAP3K7, CFLAR, BIRC3, CARD11, CSNK2A1, LCK, PRKCQ, PLCG2, SYK, IRF8	13
TNF signaling pathway	Signal transduction	1.646913221	2.801317786	5.85066243	5.985752965	4.031643564	2.986831093	PTPN11, PTK2B, MAP3K7, AKT2, CFLAR, CYLD, BIRC3, FADD, ACTB, PXN, RAC2, MAPK14, CTNNA1, NCF2, NCF1, NCF4, PLCG2, IRF8	18
VEGF signaling pathway	Signal transduction	3.035369257	2.076149727	5.560953373	8.124475524	4.350384615	4.150959379	NFATC2, PXN, RAC2, PLCG2, MAPK14	5
Fluid shear stress	Stress response	0.894291588	1.544529481	2.946340193	2.277492166	1.788583175	2.909384816	AKT2, TXN, MAP3K7, GSTM2, PDGFB, ACTG1	6
